# Cheminformatics analysis of the AR agonist and antagonist datasets in PubChem

**DOI:** 10.1186/s13321-016-0150-6

**Published:** 2016-07-08

**Authors:** Ming Hao, Stephen H. Bryant, Yanli Wang

**Affiliations:** National Center for Biotechnology Information, National Library of Medicine, National Institutes of Health, Bethesda, MD 20894 USA

## Abstract

**Background:**

As one of the largest publicly accessible databases for hosting chemical structures and biological activities, PubChem has been processing bioassay submissions from the community since 2004. With the increase in volume for the deposited data in PubChem, the diversity and wealth of information content also grows. Recently, the Tox21 program, has deposited a series of pairwise data in PubChem regarding to different mechanism of actions (MOA), such as androgen receptor (AR) agonist and antagonist datasets, to study cell toxicity. To the best of our knowledge, little work has been reported from cheminformatics study for these especially pairwise datasets, which may provide insight into the mechanism of actions of the compounds and relationship between chemical structures and functions, as well as guidance for lead compound selection and optimization. Thus, to fill the gap, we performed a comprehensive cheminformatics analysis, including scaffold analysis, matched molecular pair (MMP) analysis as well as activity cliff analysis to investigate the structural characteristics and discontinued structure–activity relationship of the individual dataset (i.e., AR agonist dataset or AR antagonist dataset) and the combined dataset (i.e., the common compounds between the AR agonist and antagonist datasets).

**Results:**

Scaffolds associated only with potential agonists or antagonists were identified. MMP-based activity cliffs, as well as a small group of compounds with dual MOA reported were recognized and analyzed. Moreover, MOA-cliff, a novel concept, was proposed to indicate one pair of structurally similar molecules which exhibit opposite MOA.

**Conclusions:**

Cheminformatics methods were successfully applied to the pairwise AR datasets and the identified molecular scaffold characteristics, MMPs as well as activity cliffs might provide useful information when designing new lead compounds for the androgen receptor.

**Electronic supplementary material:**

The online version of this article (doi:10.1186/s13321-016-0150-6) contains supplementary material, which is available to authorized users.

## Background

As one of the largest publicly accessible databases for chemical structures and their bioactivities, PubChem [1], hosted by the National Center for Biotechnology Information (NCBI), National Institutes of Health (NIH), has become an increasingly important platform to the scientific community for data sharing. With three interconnected databases: PubChem Substance (identifier SID), PubChem BioAssay (identifier AID) and PubChem Compound (identifier CID), PubChem offers open access to over 50,000 users daily via the NCBI Entrez system, as well as web-based and programmatic tools. In addition, PubChem is closely integrated with literature and other biomedical databases such as PubMed, Protein, Gene, Structure, Biosystems and Taxonomy [2]. According to the recent review [2], PubChem has been successfully applied to various fields, such as developing secondary resources and tools, studying compound-target network and drug polypharmacology, generating and validating machine learning models, and identifying lead compounds etc.

Despite of a number of previous data mining efforts [3–7], the demand only becomes higher for researchers to collectively analyze bioactivity data to solve or provide insights into scientific questions, especially in the medicinal chemistry filed, where one of the main tasks is to identify and optimize lead compounds towards desired biological activities. Thus, many researchers have attempted different computational approaches to accomplish such tasks including virtual screening based on PubChem bioactivity data [8] using the maximum unbiased validation datasets, predicting adverse drug reactions using PubChem bioassay data [9] and many others [10–13]. However, most of the studies mainly focused on the datasets with the single endpoints. With the increase in volume for the deposited data in PubChem, the diversity and wealth of information content also grows. PubChem contains hundreds of large scale high-throughput screening (HTS) projects, which often tested a common compound library providing great opportunities for bioactivity profiling research. Recently, the Tox21 program compiled a library of 10,000 compounds, and systematically carried out HTS projects against a group of targets and pathways, such as androgen receptor (AR), estrogen receptor (ER), retinoic acid receptor (RAR) and other receptors, searching simultaneously for agonists and antagonists in a pairwise manner. Data generated by these projects were deposited in PubChem. Analysis of such pairwise bioactivity data regarding to different mechanism of actions (MOA) for the same target may result in interesting discoveries, in particularly when to combine with prior data in PubChem. However, to the best of our knowledge, little work has been reported from cheminformatics study for these datasets. Thus, to fill the gap, we performed a comprehensive study focusing on this data collection using several cheminformatics methods, including scaffold analysis, matched molecular pair (MMP) analysis and activity cliff analysis.

In fact, previous studies have successfully applied such cheminformatics methods to the analysis of bioactivity data in public databases. For example, Hu and Bajorath [14] performed scaffold analysis for the DrugBank database [15] and the ChEMBL database [16]. They concluded that many drugs contain unique scaffolds with varying structural relationships to scaffolds of currently available bioactive compounds. The same authors also explored the scaffold universe of kinase inhibitors with respect to different activities [17]. Kramer et al. [18] performed matched molecular pair analysis by comparing the ChEMBL data and Novartis data suggesting that MMP analysis is a very robust tool for lead optimization and will have growing importance in daily medicinal chemistry practice. Using the ChEMBL database, Dimova et al. [19] presented a systematic evaluation of activity cliff progression in evolving compound datasets. They found that activity cliffs currently are not a major focal point of practical medicinal chemistry efforts and anticipated that chemically unexplored activity cliffs should provide significant opportunities for further study in medicinal chemistry. All these findings indicate that cheminformatics studies are playing important roles in medicinal chemistry. However, it can be noted that most of such studies are mainly focusing on the ChEMBL database.

In this work, we performed a comprehensive cheminformatics study for the Tox21 assay data deposited in the PubChem database to investigate the molecular scaffold characteristics, matched molecular pairs as well as activity cliff in the individual target-based dataset (i.e., either AR agonist dataset or antagonist dataset). Moreover, we also performed a computational analysis for the combined dataset (i.e., commonly tested compounds) between the AR agonist and antagonist datasets in Tox21. Several interesting observations are reported and discussed.

## Material and experimental methods

### Bioassay data

Bioactivity data for the agonist and antagonist screens for the androgen receptor (AR, GenBank: AAI32976.1) were retrieved from the PubChem BioAssay database. For the agonist screen (AID 743053), there were 372 substances reported as active outcomes and 9070 substances as inactive outcomes from a total of 10,486 substances, while for the antagonist screen (AID 743063), 670 substances were reported as active and 7770 substances as inactive from the same compound library. These original compounds were subject to further filtering as described below.

### Preprocessing of the original data

To obtain the final dataset for analysis, the following steps were applied: (1) compounds with missing readouts were removed (original 8, 111 unique CIDs were reduced to 8110 for both the AR agonist and antagonist datasets); (2) redundant compounds (same CIDs and same readouts but different SIDs) were removed (CIDs remained the same for both the AR agonist and antagonist datasets); (3) compounds with discrepant bioactivity, meaning the same chemical structure (CID) with contradictory bioactivity report (same CIDs but different readouts and different SIDs), were removed (CIDs were reduced to 7866 for the AR agonist dataset, and 7678 for the AR antagonist dataset, respectively); (4) compounds without outcome annotations of “Active” and “Inactive” were removed (CIDs were reduced to 7174 for the AR agonist dataset, and 6321 for the AR agonist dataset, respectively); (5) compounds of mixtures were removed (CIDs were reduced to 5649 for the AR agonist dataset, and 4956 for the AR antagonist dataset, respectively); and (6) compounds containing no ring-like structures were removed (CIDs were reduced to 4162 for the AR agonist dataset, and 3563 for the AR antagonist dataset, respectively). Finally, the PubChem CID (representing unique chemical) rather than SID (representing a sample) was used as the compound identifier for keeping data consistency. The final AR agonist dataset consisted of 172 “Active” molecules and 3990 “Inactive” ones, and the AR antagonist dataset consisted of 322 and 3241 of “Active” and “Inactive” compounds, respectively. The R software [20] was used to perform the analysis.

### Scaffold construction

A molecular scaffold, according to the definition introduced by Bemis and Murcko [21], is often called BM scaffold, which is extracted from the molecule by removing all substituents while retaining aliphatic linkers between ring systems. In this work, the scaffolds of the AR agonist and antagonist datasets were constructed by using the method proposed by Matlock et al. [22]. Specifically, the scaffold network generator (sng) tool [22], taking the input of SDF format of molecules, was used to generate the molecular scaffolds. In addition, each scaffold was also reduced to an even more brief molecular framework (also called cyclic skeleton (CSK) [23]) by converting all heteroatoms to carbon and turning all bonding orders (double bonds or triple bonds) to one. Therefore, each CSK represents a series of topologically equivalent scaffolds. The RDKit software [24] was used to obtain the CSKs from the corresponding scaffolds.

### Matched molecular pair

As described by Hussain and Rea [25], an MMP is a pair of molecules that only differ by a structural change at a single site, which has become a major tool for analyzing large chemistry dataset for promising chemical transformations [18]. In this work, size-restricted MMPs were constructed to limit structural differences between compounds to small replacements as reported previously [26], which was done in the following procedures: (1) the invariant core fragment was required to have at least twice as the size of each exchanged fragment; (2) the maximal size of an exchanged fragment was limited to 13 non-hydrogen atoms and (3) the size difference between two exchanged fragments was set to eight atoms as the maximum. Thus, the generated MMPs provided a conservative measure of structural similarity [23]. All MMP calculations were calculated using the algorithm proposed by Hussain and Rea [25]. Specifically, the *mmpa* module implemented in RDKit software [24] was used to generate the MMPs. The module was ran with the default settings except the maximal size change in heavy atoms allowed in MMPs identified (13 in this work). The other steps were performed using the R software [20], which took the SMILES format of molecules as input.

### Activity cliff

A common definition for activity cliff is that a pair of structurally similar molecules exhibit a large difference in bioactivity potency [27]. For the similarity measures between molecules, different methods have been successfully applied, whereas Tanimoto similarity based on various fingerprint descriptors (e.g., PubChem fingerprints, MACCS fingerprints, ECFP4 fingerprints and many others [27]) and MMP-based similarity are among the most popular ones [28]. In this work, the latter was adopted. In addition, the PubChem bioactivity outcome annotations (i.e., active or inactive) provided by depositors were directly used to obtain the bioactivity potency differences. Thus, the generated activity cliffs herein were MMP-based cliffs.

## Results and discussion

As one of the nuclear hormone receptors, AR (GenBank: AAI32976.1) plays a critical role in AR-dependent prostate cancer and other androgen related diseases. Several endocrine disrupting chemicals and their interactions with AR may cause disruption of normal endocrine function as well as interfere with metabolic homeostasis, reproduction, developmental and behavioral functions. Thus, in order to identify the agonists and antagonists of AR signaling, GeneBLAzer AR-UAS-bla-GripTite cell line containing a beta-lactamase reporter gene under control of an upstream activator sequence stably integrated into HEK293 cells was used to screen the Tox21 10K compound library. In this work, we have investigated the screened compounds by applying several cheminformatics methods in order to mine useful information for the design of lead compounds.

### Scaffolds and CSKs of the AR agonist and antagonist datasets

**Fig. 1:**
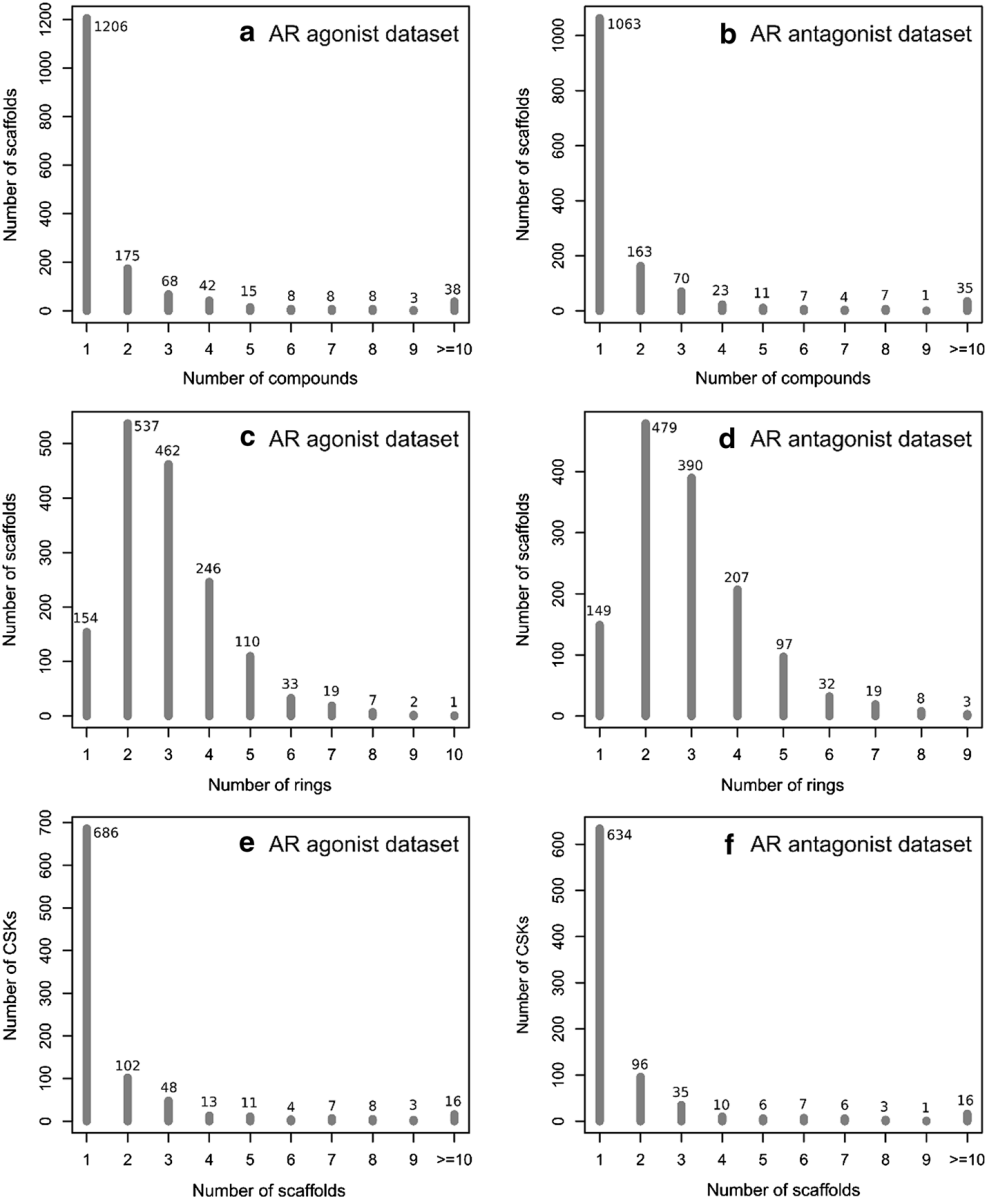
Frequency of scaffolds that cover a certain number of compounds for the agonist dataset (**a**) and antagonist dataset (**b**); frequency of scaffolds that have a certain number of rings for the agonist dataset (**c**) and antagonist dataset (**d**); frequency of CSK that cover a certain number of scaffolds for the agonist dataset (**e**) and antagonist dataset (**f**) of AR

After applying the filtering criteria described in the method section, the compounds used in the analysis including both the AR agonist and antagonist datasets are listed in Table 1, together with statistics for scaffolds and CSKs. As we can see that we finally obtained a total of 4162 compounds from the PubChem Tox21 agonist dataset (AID 743053) containing 172 active and 3990 inactive ones to perform further research. It should be noted that each compound possesses a unique CID indicating that it has a distinct chemical structure. On the contrary, the AR antagonist dataset (AID 743063) includes relative less unique compounds (3563) but with more active ones of 322 and less inactive ones of 3241 compared to the AR agonist dataset. In order to explore the building blocks or core structures of these compounds of different mechanism of actions, which are of high interest to pharmaceutical research, we performed scaffold analysis. Here, the scaffold refers to the popular BM-scaffold. On the basis of these identified 4162 compounds in the agonist dataset, we extracted 1571 unique scaffolds. Thus, each scaffold on average represents about 2.6 compounds. It is also noted that there are about 77 % scaffolds which are only found in a single compound. Among the scaffolds, benzene represents the most compounds. In this case, one benzene scaffold represents 1147 compounds, followed by the pyridine scaffold representing 67 compounds. These findings indicate that the series of compounds tested in the AR dataset are structurally diverse. Figure 1a shows the distribution of the compounds among the identified scaffolds for the AR agonist dataset. Furthermore, we also would like to examine the distribution of rings in these scaffolds. As shown in Fig. 1c, it is evident that most of the scaffolds consist of two or three rings (64 % of the whole scaffolds). For the AR antagonist dataset, 3563 compounds are covered by 1384 scaffolds. Among them, 1063 scaffolds (about 77 % of the whole scaffolds) show a one-scaffold-one-compound relationship again with benzene and pyridine as the most common ones. Figure 1c shows the distribution of the compounds among the scaffolds for the AR antagonist dataset. While exploring the number of rings related with scaffolds, it can be noted that most scaffolds (63 %) have two or three rings which is the same as the AR agonist dataset, but the maximum number of rings is 9 rather than 10 compared to the corresponding agonist dataset (Fig. 1d). Based on this analysis, it can be noticed that the studied compounds are ring-less and diverse.

**Table 1:** Summary of the studied AR agonist and antagonist datasets

	Agonist			Antagonist		
	Total	Active	Inactive	Total	Active	Inactive
Number of unique compounds	4162	172	3990	3563	322	3241
Number of unique scaffolds	1571	72	1521	1384	198	1248
Number of unique CSKs	895	53	865	814	160	717
Diversity index	–	0.50	0.66	–	0.61	0.67

It is well known that datasets from HTS have the imbalanced nature, which means that the majority of screened compounds exhibit inactive outcomes, while just a minority part of them show active outcomes. In our study, the inactive compounds of the AR agonist dataset are more than 23 folds larger than the active ones. By comparing the scaffolds of them, the former are more than 21 folds of the latter (Table 1). However, one can notice that the imbalanced ratio between the inactive and active CID counts, and that between the scaffold counts for the compounds of the AR antagonist dataset are relatively low compared to those of the agonist dataset, which are about 10 and 6 for the compounds and scaffolds, respectively, which indicates that the identified agonists are more structurally specific while the antagonists are rather structurally diverse in this studied datasets. By calculating the diversity index (DI) [29] of active and inactive molecules, using the PubChem fingerprints for the AR agonist dataset, it can be noticed that the DI of active compounds is 0.50, which is relatively less than the inactive DI of 0.66 though the number of former dataset is largely less than the latter. For the AR antagonist dataset, the DIs are 0.61 and 0.67 for the active and inactive compounds, respectively. The almost equal DIs indicate that the investigated datasets are diverse.

We further decomposed the scaffolds to CSKs which are used to elucidate more general skeletons of the scaffolds. According to the previously mentioned criteria, a total of 1571 scaffolds are reduced to 895 CSKs for the AR agonist dataset, where the active 72 scaffolds consist of 53 CSKs and the inactive 1521 ones consist of 865 CSKs (Table 1). Likely, the AR antagonist dataset consists of 814 unique CSKs, in which the active and inactive ones consist of 160 and 717 CSKs, respectively (Table 1). Figure 1e, f show the distribution of scaffolds among CSKs for the AR agonist and antagonist datasets, respectively. There are about 77 % of the whole CSKs in the AR agonist dataset exhibiting a one CSK to one scaffold relationship, while this ratio is 78 % for the AR antagonist dataset, again indicating the screened compound library is structurally diverse enough. The whole list can be found in the Additional file 1: Table S1.

**Fig. 2:**
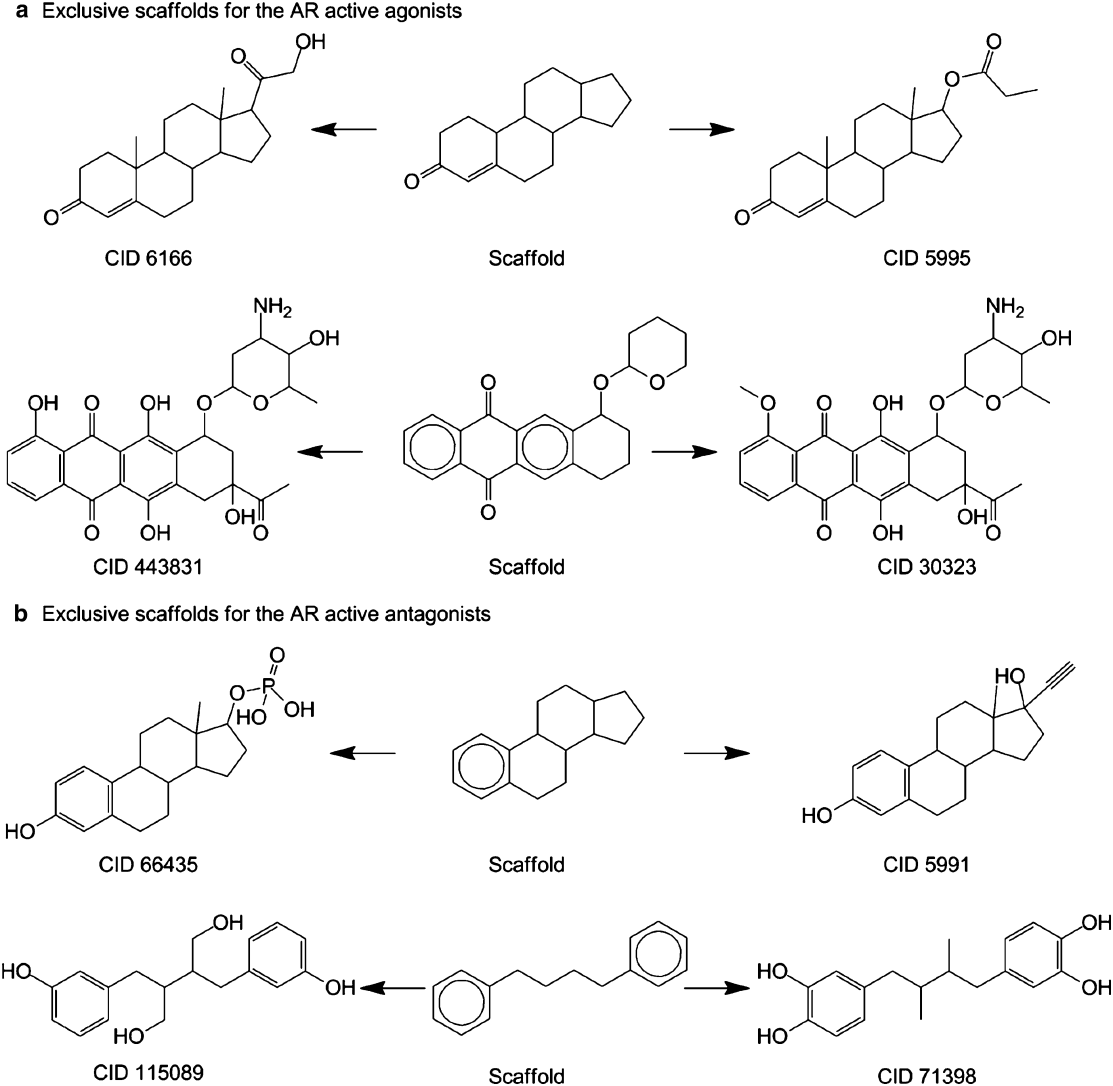
Representative exclusive scaffolds for the AR active agonists (**a**) and AR active antagonists (**b**)

More importantly, a comparison for the active and inactive scaffolds of the AR agonist dataset shows 22 overlapping scaffolds, and there are 50 scaffolds that exclusively represent only active compounds in the agonist dataset. Figure 2a gives the representative structures of these distinct active scaffolds. Besides the binary outcomes, we have also looked into the potency for these active compounds as the AR agonists. Herein, we converted the IC_50_ (uM, micromolar) as pIC_50_ (M, molar). It should be pointed out that when we extracted the potency value for each unique active compound, we also applied some criteria: (1) if the same compound has multiple potency values with the same log order, we obtained the mean value of them as the final potency value; (2) if the same compound has multiple potency values with the difference of more than one log order, we removed such compounds. Finally, 49 exclusive scaffolds were derived representing 98 unique compounds. These compounds exhibit a scale of potency values from 4.26 to 9.19 molar. It can be noticed that two compounds (CID 10631 with 4-ring scaffold “O=C1CCC2C(=C1)CCC1C2CCC2C1CCC2” named sca_1 and CID 3033968 with 4-ring scaffold “O=C1CCC2C(=C1)CCC1C2CCC2C1C=CC2” named sca_2) shows the most potency values of more than 9 molar. Both sca_1 and sca_2 represent a total of 35 unique active compounds, where the former represents the majority of 34 compounds with the potency values from 5.67 to 9.10 molar (around 79 % of them present the potency values of more than 7 molar), and the latter consists of only one compound (CID 3033968). The compounds with high potency values may provide insight for lead design. Likely, 136 scaffolds exclusively cover only active compounds of the AR antagonist dataset with the representative ones shown in Fig. 2b. When analyzing the potency values of the exclusive antagonists, we filtered out one scaffold and kept a total of 135 scaffolds representing 171 unique compounds with the potency values from 4.23 to 7.95. Eleven compounds from 9 scaffolds show the most potency values of more than 7 molar. When we investigated the activity distribution for all compounds from these 9 scaffolds, it can be noticed that there are a total of 13 compounds with the potency values from 6.65 to 7.95 molar, indicating these scaffolds represent the consistent activity distribution, though the bioactivity (7.95 molar) for the most potent antagonist is two orders lower compared to that of the strongest agonists (9.19 molar). Such exclusive scaffolds should be explored further for lead compound development with optimal potency and selectivity. More information about the exclusive active scaffolds for the AR agonists and antagonists can be found in the Additional file 2: Table S2.

### MMPs and activity cliffs of the AR agonist and antagonist datasets

**Fig. 3:**
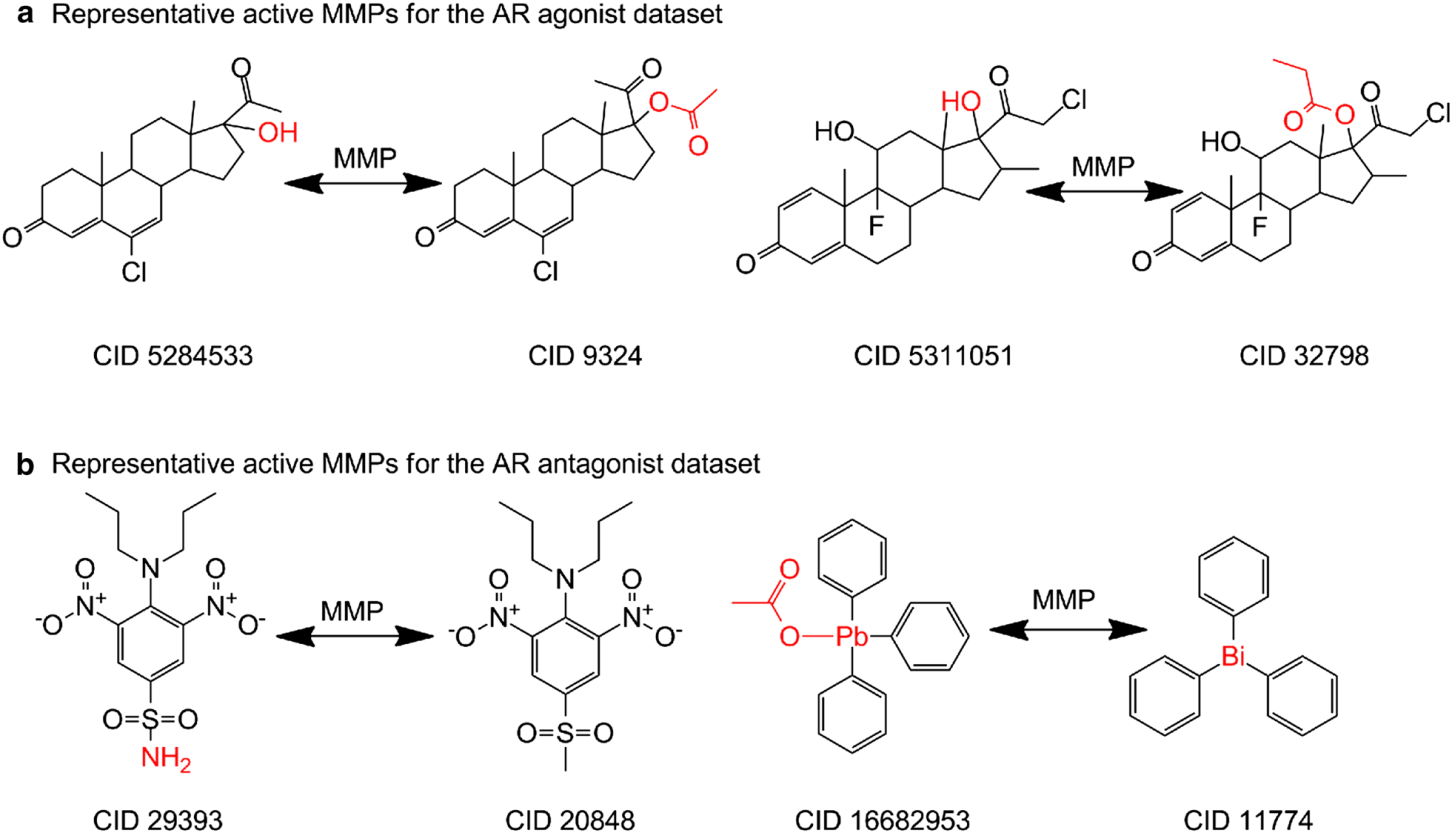
Representative active MMPs for the AR agonists (**a**) and AR antagonists (**b**)

**Fig. 4:**
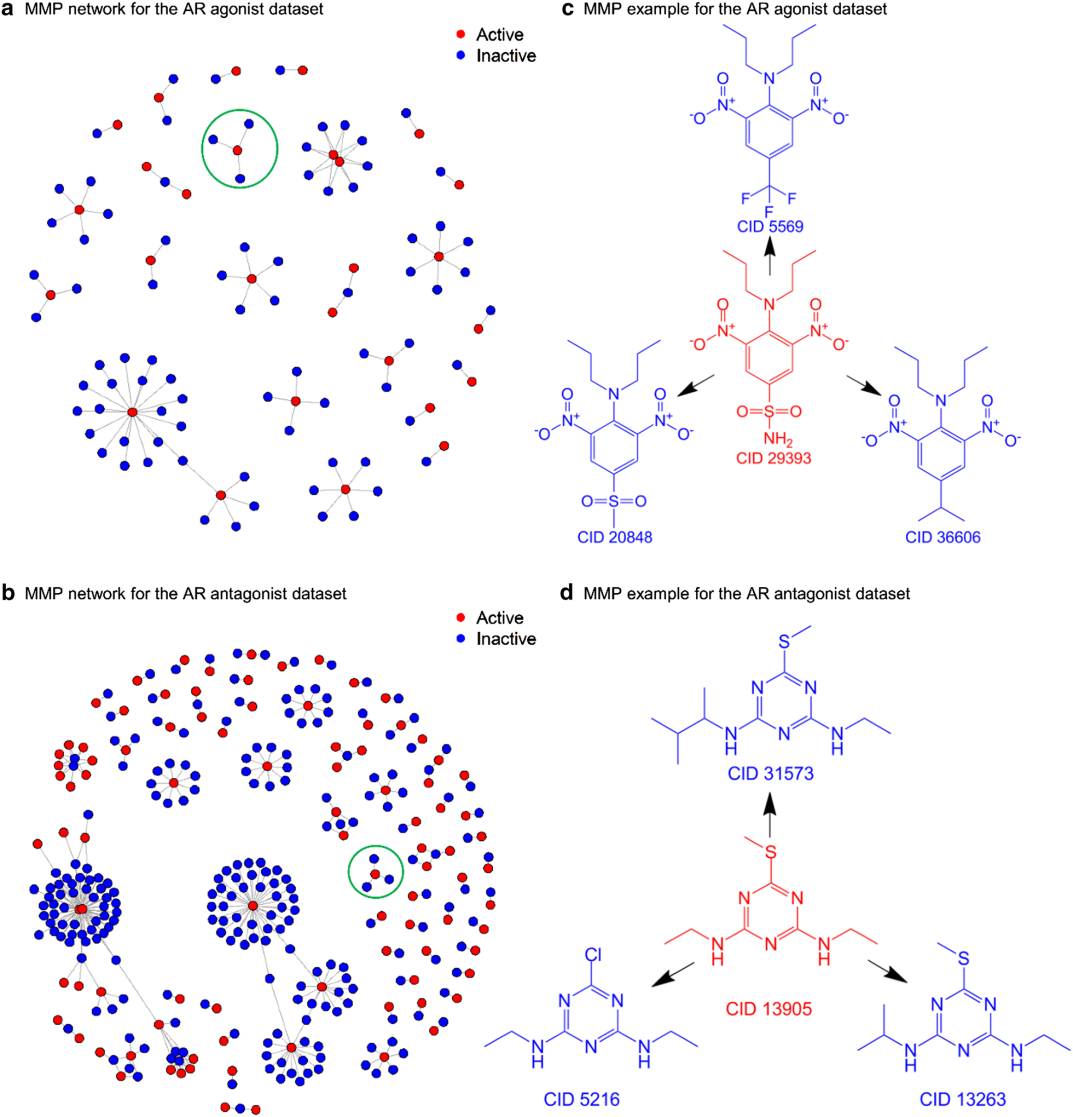
MMP network for the AR agonist dataset (**a**); MMP network for the antagonist dataset (**b**); MMP example surrounded by *green circle* for the AR agonist dataset (**c**); MMP example surrounded by *green circle* for the AR antagonist dataset (**d**)

Matched molecular pair (MMP) analysis has become a standard tool for the extraction of medicinal chemistry knowledge from large databases [18]. In addition, MMP formalism is descriptor-independent, metric-free and chemically intuitive [26], which motivated us to perform MMP analysis based for the AR datasets. For the agonist dataset, we accomplished MMP calculations from the original 4162 molecules according to the rules described in the method section. Herein, it should be pointed out that a pair of compounds may generate multiple MMPs. In such case, we retained only one of the MMPs by applying the additional selection rules. First of all, we calculated the absolute deviation of the heavy atom count between the exchanged groups, and retained the pair with the smallest deviation value. Secondly, if there still exists multiple pairs with the same smallest deviation value, we randomly chose one of such pairs. As a result, 9695 MMPs were generated to satisfy the specified criteria. By classifying all MMPs, one can notice that the MMPs with the same bioactivity outcomes are 9603 including the inactive MMPs of 9462 and the active MMPs of 141. Herein, an inactive MMPs refer that the ‘left’ molecule and ‘right’ molecules in a pair show both inactive outcomes according to the bioactivity annotation depositors provided, and this is the same for an active MMPs with both molecules in the pair being active compounds. Moreover, a total of 92 MMPs are observed with the molecule pairs associated with opposite bioactivity outcomes (i.e. with one of the molecule reported as active, and the other one in the pair as inactive) for the AR agonist dataset, indicating potential activity cliffs which will be further discussed in the following section. For the AR antagonist dataset, we obtain a total of 8049 MMPs from the original 3563 molecules. Among them, 7717 MMPs with the same outcomes consist of 7623 inactive MMPs and 94 active MMPs. Furthermore, 332 MMPs consist of molecule pairs with opposite bioactivity outcomes. Table 2 shows the summary of the generated MMPs for the AR agonist and antagonist datasets, respectively. In this series of generated MMPs, one may be first interested in the active MMPs to give insight into property optimization for the compounds such as improving solubility, oral availability, protein binding, and so forth [30]. Figure 3 shows several representative active MMPs for the AR agonist and antagonist datasets, separately. The whole networks for both datasets are shown in Fig. 4. From this figure, one can see that most active compounds are used as hubs to connect the inactive ones in the generated pairs, indicating that more attention should be paid when designing new lead compounds based on these hub compounds since analogs may be located at the bottom of the activity cliff.

**Table 2:** MMPs for the AR agonist and antagonist datasets

Category	Number of MMPs		Outcome pattern	
	Agonist	Antagonist	Left molecule	Right molecule
Inactive MMPs	9462	7623	Inactive	Inactive
Active MMPs	141	94	Active	Active
Activity cliff MMPs	92	332	Inactive	Active

**Fig. 5:**
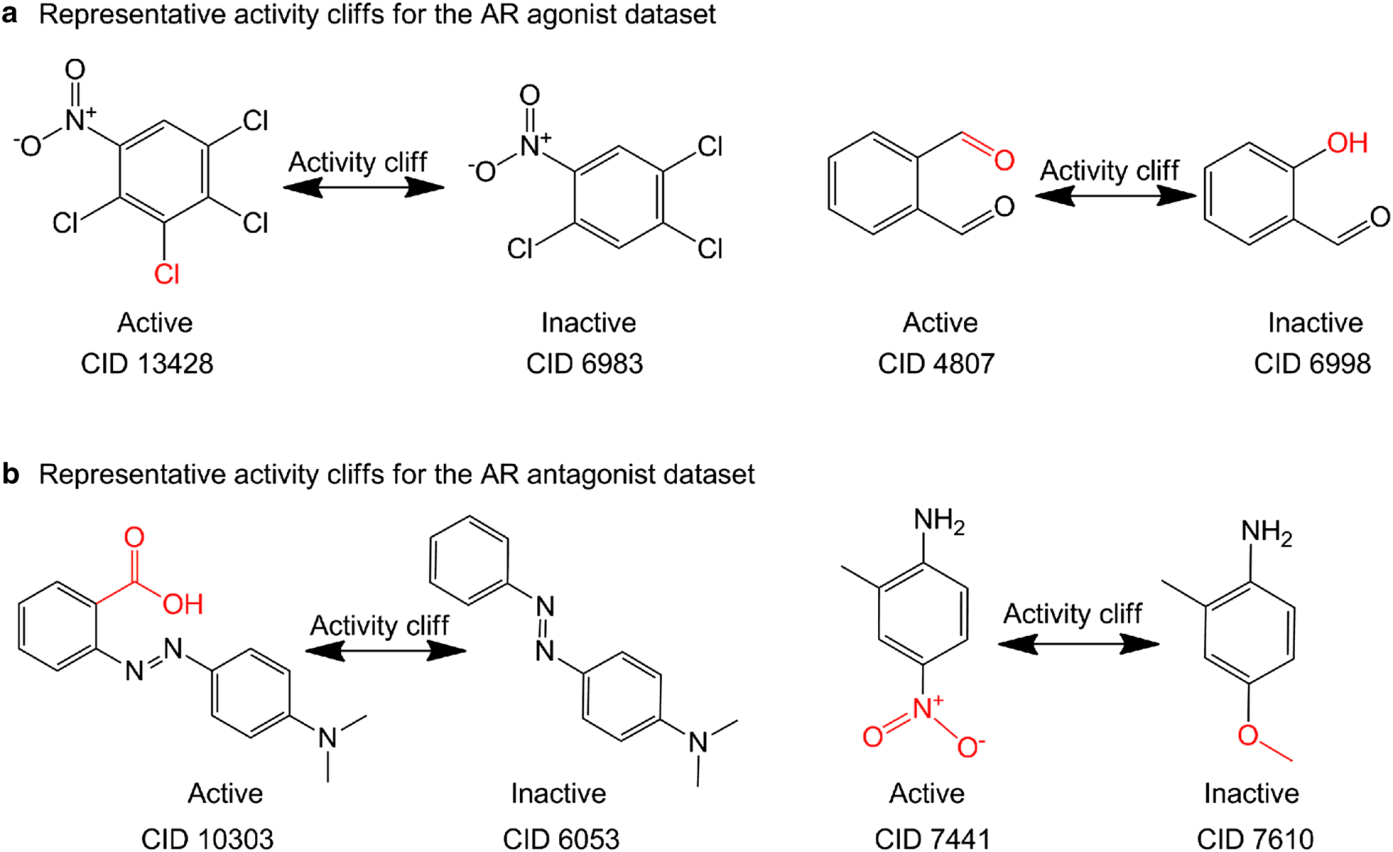
Representative MMP-based activity cliffs for the AR agonist dataset (**a**) and AR antagonist dataset (**b**)

In addition to MMP recognition, activity cliff analysis has been another critical approach for medicinal chemistry research, for which activity cliffs are often encountered in hit-to-lead projects. Activity cliffs represent centers of SAR discontinuity in activity landscapes of compound datasets and are focal points of SAR exploration [31]. It is also worthy to point out that activity cliffs fall out of the similarity-property principle and are usually incorrectly predicted by quantitative structure–activity relationship models [27]. Given the importance of activity cliff analysis in medicinal chemistry, several studies have been reported mainly based on the ChEMBL database [19, 32–34]. To gain insight for lead identification and optimization, we analyzed MMP-based activity cliffs for both Tox21 AR agonist and antagonist datasets. We used the binary bioactivity outcome annotations, e.g. active versus inactive, provided in the dataset submissions as the corresponding activities. As shown in Table 2, we identified 92 MMP-based activity cliffs for the AR agonist dataset, while for the AR antagonist dataset, 332 MMPs with potential activity cliffs are observed. Such activity cliffs are of high interest and can be valuable to medicinal chemists for lead compound design and development. Figure 5 shows the representative MMP-based activity cliffs for the AR agonist and antagonist datasets, respectively. The whole active MMPs list is provided in the Additional file 3: Table S3.

### Mechanism of actions analysis

In addition to the activity cliff analysis within the respective AR agonist dataset and antagonist dataset, we also carried out MMP-based analysis by combing the agonist and antagonist datasets taking the advantage that both screens tested the same compound library. We compiled a total of 3293 such common compounds for both datasets. We first removed those compounds (3008) with inactive outcome in both of the AR agonist and antagonist datasets as we attempted to focus on the compounds with potential agonist and antagonist function as identified in the two screens. As a result, the remaining 285 compounds with pairwise mechanism of actions (i.e. agonist vs. antagonist) were applied to further study with two questions in mind: (1) to check structure-based bioactivity overlap; and (2) to explore MMP-based MOA cliffs.

**Fig. 6:**
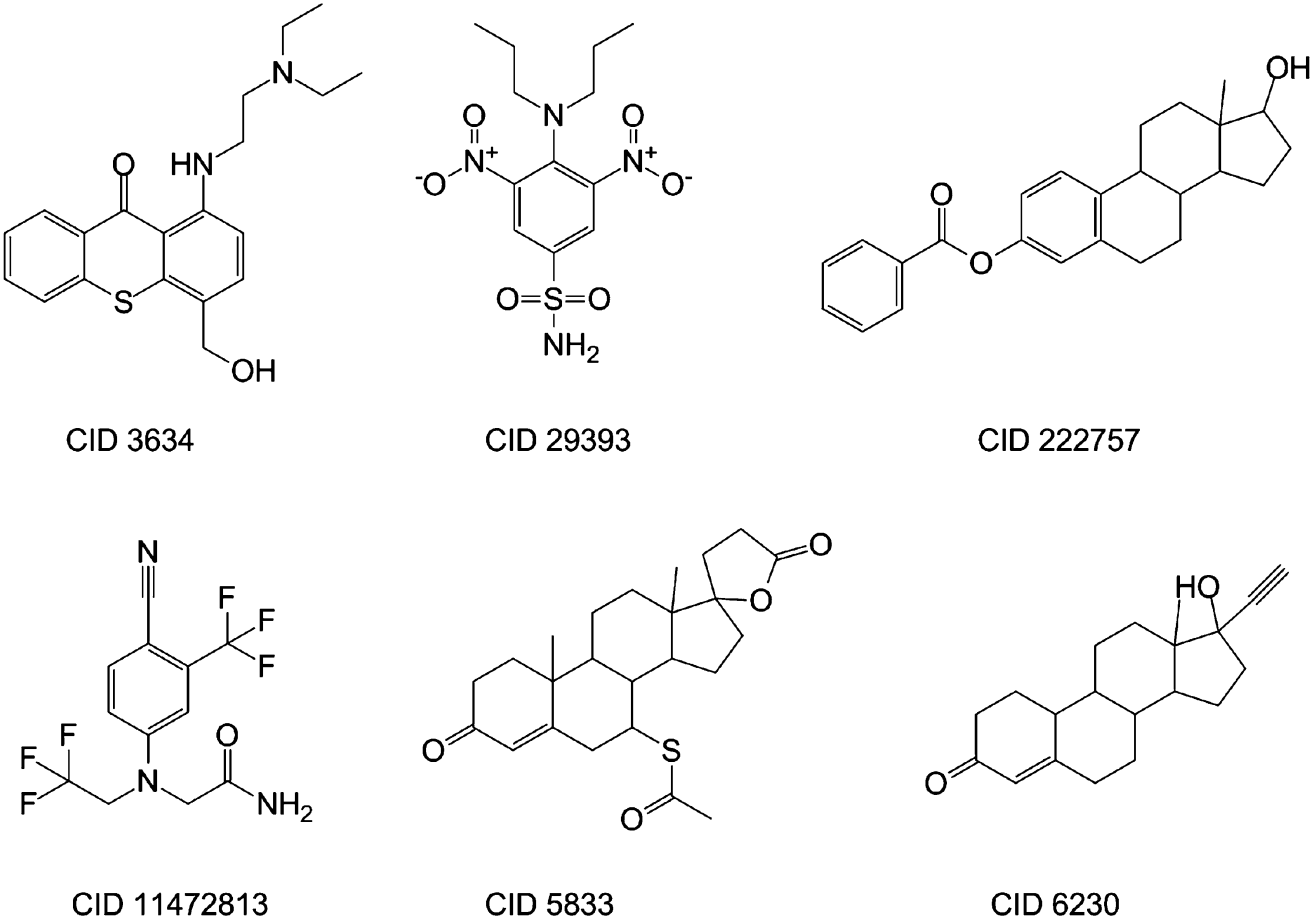
Representative molecular structures for the dual action molecules

To answer the first question, we organized the 285 common compounds according to their annotated bioactivity outcomes. It can be noticed that 240 molecules exhibited opposite outcomes (i.e., they are either agonists or antagonists of AR). On the other hand, and surprisingly, 45 compounds (Additional file 4: Table S4) were reported as active in both screens. This finding is interesting since it means that these 45 molecules were recognized as both agonists and antagonists of AR simultaneously, which may be explained by two folds: (1) they indeed possess both MOA detected by different screens; (2) this observation may reflect underlying experimental errors. In any case, further experimental investigation is needed to confirm this finding. Figure 6 shows the representative structures for these 45 compounds with dual MOA reported.

**Fig. 7:**
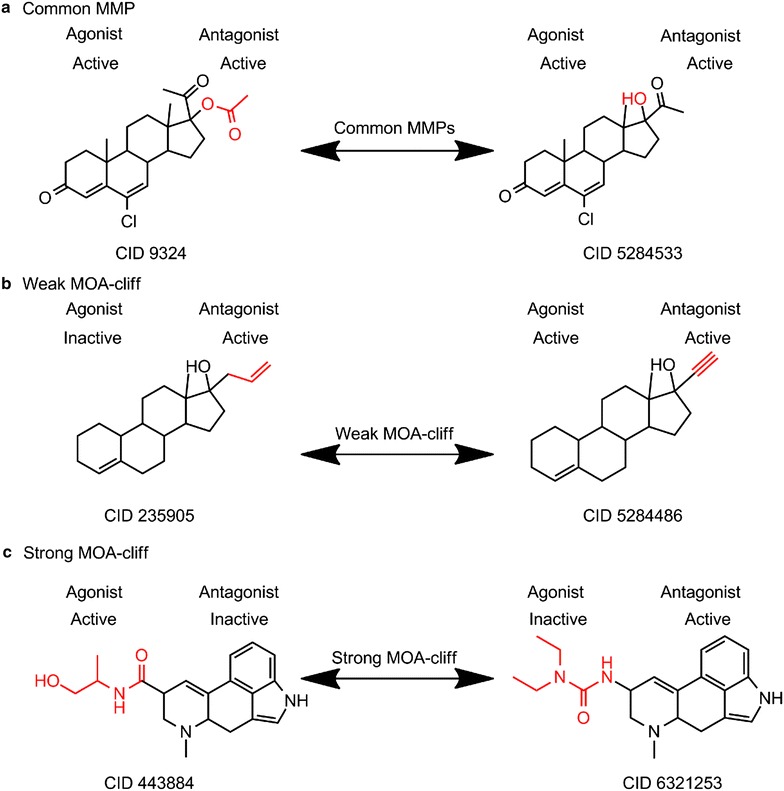
Representative MMP (**a**), weak MOA-cliff (**b**) and strong MOA-cliff (**c**) for the combined dataset

For the second question, MMP analysis was performed for the 285 common compounds. As a result, a total of 78 MMPs were obtained after applying above-mentioned filters. We classify these 78 MMPs into 3 categories as shown in Table 3. The largest category has 64 MMPs, where both molecules in a pair show the same MOA, and we call it same MOA. Interestingly, the remaining 14 MMPs form MOA-cliffs, a novel concept we proposed, which refers to that a pair of structurally similar molecules present different MOA. Among the 14 MOA-cliffs, there are 13 MMPs to be considered as weak MOA-cliffs because they just show partly different MOA. Compared to the weak MOA-cliffs, it is very interesting to observe a strong MOA-cliff, which presents a totally opposite MOA between the molecules (CID 443884, AR agonist, 4.69 molar; CID 6321253, AR antagonist, 4.77 molar) (Table 3). It is true that both molecules show the relatively less potency values, but the outcome annotations from the depositor indeed elucidate them as agonist and antagonist, respectively. It should also be pointed out that by comparing CID 443884 with CID 6321253, the exchanged fragments are slightly different where the tail of former molecule shows the more polar characteristic than the latter one. That may be a possible reason why they show the opposite MOA. Figure 7 exhibits the representative structural pairs of the identified MMPs and MOA-cliffs for the combined dataset (the whole list can be found in the Additional file 5: Table S5). Despite of the high interest for this observation, it should be pointed out the bioactivities of the compounds would need to be verified by further investigations. Regardless, the analysis indicates that the cheminformatics tools may be used to provide in-depth analysis of big chemical biology data, to understand the relationship between chemical scaffolds, structures and their biological functions, and in particularly to recognize interesting compound pairs that demonstrate completely different mechanism of actions, hence to provide guidance for further medicinal chemistry study. Indeed, there are more datasets from the Tox21 program and other HTS projects with data available in PubChem screened for both agonists and antagonists, or activators and inhibitors against a target, which will be subject to future study.

**Table 3:** Summary of MMPs and cliffs for the combined AR dataset

MOA pattern	Number of MMPs		Left molecule		Right molecule	
			Agonist	Antagonist	Agonist	Antagonist
Same MOA	64	17	Active	Active	Active	Active
		26	Inactive	Active	Inactive	Inactive
		21	Active	Inactive	Active	Inactive
Weak MOA-cliffs	13	7	Inactive	Active	Active	Active
		6	Active	Active	Active	Inactive
Strong MOA-cliffs	1	1	Active	Inactive	Inactive	Active

## Conclusions

In this work, we analyzed the pairwise agonist and antagonist AR data including scaffold analysis, matched molecular pair and activity cliff. Scaffolds with distinct agonist or antagonist bioactivity as well as those showing activity cliffs were identified. In addition to the activity cliffs regarding to a single MOA, we also carried out activity cliff analysis by combing the AR agonist and antagonist datasets. We proposed a novel MOA-based cliff concept to indicate a pair of structurally similar molecules which exhibit the opposite MOA. In a summary, by a thorough investigation of the Tox21 AR datasets, a series of scaffolds, MMPs, activity cliffs as well as MOA-cliffs have been identified or proposed. We hope this analysis might be helpful for optimizing or designing novel AR agonists and antagonists, and to find key structure elements for determining mechanism of actions for small molecule compounds.

## Additional files

10.1186/s13321-016-0150-6 The whole list of studied AR agonist and antagonist datasets.
10.1186/s13321-016-0150-6 Exclusive active scaffolds for the AR agonists and antagonists.
10.1186/s13321-016-0150-6 The whole list of active MMPs for the AR agonist and antagonist datasets.
10.1186/s13321-016-0150-6 Compounds reported as active in both AR agonist and antagonist screens.
10.1186/s13321-016-0150-6 The whole list of structural pairs of the identified MMPs and MOA-cliffs for the combined dataset.
